# Habitual Physical Activity and Walking Endurance Following Inpatient Rehabilitation in Children With Cerebral Palsy

**DOI:** 10.7759/cureus.109862

**Published:** 2026-05-29

**Authors:** Pawel Chmara, Amadeusz Brzykcy, Sabina Brazevic, Marek Jozwiak

**Affiliations:** 1 Department of Pediatric Orthopedics and Traumatology, Wiktor Dega Orthopedic and Rehabilitation Clinical Hospital of Poznan University of Medical Sciences, Poznań, POL; 2 Student Scientific Society, Poznan University of Medical Sciences, Poznań, POL

**Keywords:** cerebral palsy, gmfcs, inpatient rehabilitation, physical activity, walking endurance

## Abstract

Background: Cerebral palsy (CP) at GMFCS level II is the most functionally heterogeneous ambulatory group, with high intra-group variability in walking endurance and daily physical activity. The relationship between six-minute walk test (6MWT) outcomes and accelerometer-based monitoring following inpatient rehabilitation remains unclear.

Aim: The aim of this study was to assess correlations between wear-time-normalised habitual physical activity (PA) parameters and rehabilitation-induced change in walking endurance, and to determine whether objectively measured spatiotemporal gait parameters explain the inverse relationship between metabolic equivalent of task (MET)-based PA classification and baseline 6MWT performance in this population.

Methods: Twenty-five children with CP at GMFCS II (female: 10, male: 15; age: 13.40 ± 1.12 years) completed a two-week inpatient rehabilitation programme. The 6MWT was performed on the first and final day. PA was monitored over 72 hours using a wearable device, which was removed during sleep, bathing, and activities that might cause damage to the device; data were not recorded during structured therapy sessions. Participants were classified as active (n = 13) or inactive (n = 12). Analysis included paired t-test, Pearson and Spearman correlations, Mann-Whitney U test, and Cohen's d_z_. Spatiotemporal gait parameters were obtained from instrumented gait analysis.

Results: Significant improvement in 6MWT distance was observed in the entire group (moderate effect), and the active subgroup (medium-to-large effect); no improvement was found in the inactive subgroup. No significant correlations between normalised PA measures and 6MWT improvement were identified. At baseline, the active subgroup showed lower 6MWT distance despite higher step accumulation. Between-group PA differences were significant. Spatiotemporal analysis revealed inefficient gait biomechanics in the active subgroup (lower walking speed, shorter step and stride length, longer stance phase). An exploratory analysis found a negative correlation between baseline 6MWT and rehabilitation-induced change in the inactive subgroup.

Conclusions: Two weeks of inpatient rehabilitation produced clinically meaningful improvements in walking endurance, particularly in the active subgroup. Habitual PA intensity and endurance gains were not correlated. Inefficient gait biomechanics in the active subgroup may help explain the paradoxical inverse relationship between MET classification and baseline 6MWT performance.

## Introduction

Cerebral palsy (CP) is the most common cause of physical disability in childhood, defined as a group of permanent disorders of movement and posture resulting from non-progressive disturbances occurring in the developing fetal or infant brain [1]. The Gross Motor Function Classification System (GMFCS) is the gold-standard tool for stratifying the severity of motor impairment and guides clinical decision-making, research design, and prognosis [2]. The GMFCS classifies children with CP into five levels based on self-initiated movement, movement quality, and assistive technology needs, ranging from unrestricted walking (level I) to full dependence on a manual wheelchair (level V).

Children classified at GMFCS level II walk without assistive devices indoors but may require support on uneven surfaces or for longer distances. This functional level is characterised by substantial heterogeneity; some children approach the motor repertoire of GMFCS I, while others exhibit walking patterns and energy expenditure profiles closer to GMFCS III [3]. Crucially, GMFCS level II is associated with a documented risk of functional deterioration during the pubertal growth spurt, making early and sustained intervention a clinical priority [4]. Inclusion of a single GMFCS level reduces between-subject variability in motor impairment severity, allowing detection of within-level relationships between habitual physical activity and walking endurance.

Physical activity (PA) plays a fundamental role in maintaining and improving functional endurance in children with CP, who are significantly less active than their typically developing peers, spending more time in sedentary behaviour and accumulating fewer steps per day [5].

Wearable accelerometers enable objective, continuous quantification of habitual physical activity in free-living conditions, capturing parameters such as step count, activity duration, and estimated energy expenditure. Such devices have been validated for use in children with CP and represent a clinically practical alternative to laboratory-based assessment [6]. Despite the clinical importance of both objective PA monitoring and the six-minute walk test (6MWT) assessment, little is known about whether habitual PA levels, as quantified by accelerometer-based monitoring, predict or correlate with the magnitude of improvement in walking endurance following structured inpatient rehabilitation. While relationships between PA and GMFCS have been examined longitudinally in CP [5,6], and accelerometer-based monitoring has been validated as an outcome measure in this population, the specific question of whether pre-rehabilitation habitual activity intensity predicts 6MWT gains following inpatient rehabilitation has received limited attention.

One previous study examined this question using the same accelerometer protocol and 6MWT design in a sample of children with CP at GMFCS II [7]. The earlier study found a significant improvement in walking endurance in the active subgroup but not in the inactive group and noted a paradoxical finding: children classified as physically inactive achieved substantially greater 6MWT distances than their active peers. The authors called for studies incorporating spatiotemporal gait analysis to explain this inverse relationship. The present study was designed to address this gap by enrolling a larger group and incorporating instrumental gait analysis to test the hypothesis that metabolic equivalent of task (MET)-classified active children exhibit inefficient gait biomechanics, explaining their paradoxically lower walking endurance despite higher step accumulation.

The aims of this study were to assess the correlation between wear-time-normalised habitual physical activity parameters (step rate, caloric expenditure per hour, and percentage of active wear time) and the rehabilitation-induced change in walking endurance in children with CP at GMFCS II, and to determine whether spatiotemporal gait parameters explain the inverse relationship between MET-based physical activity classification and baseline (T1) walking endurance observed in this population.

## Materials and methods

This study was conducted at the Department of Paediatric Orthopaedics and Traumatology, Poznań University of Medical Sciences, Poznań, Poland. It was approved by the Bioethics Committee at the Karol Marcinkowski Medical University in Poznań (resolution number: 633/20). Physical activity was monitored over a 72-hour period using the McRoberts DynaPort MoveMonitor (McRoberts B.V., Hague, Netherlands), a lumbar-mounted multi-sensor wearable device (dimensions: 106.6 × 58 × 11.5 mm; weight: 55 g; 1 GB flash memory; sensors: triaxial accelerometer, triaxial magnetometer, barometer, thermometer) [8]. Participants were instructed to wear the device throughout the day, removing it only during sleep, bathing, and activities that might cause damage to the device. The minimum valid wear time was set at 16 hours. 

Study population

Twenty-nine children with CP at GMFCS level II were initially recruited (female: 12; male: 17; mean age: 13.48 ± 1.09 years, range 12-16). Four were subsequently excluded due to insufficient accelerometer wear time (< 16 hours over the monitoring period), leaving a final analysis sample of 25 children (female: 10; male: 15; mean age: 13.40 ± 1.12 years, range 12-16). Participants were classified as active or inactive using the device’s MET-based activity-intensity output, which categorises activity as sedentary, moderate, and vigorous and compares accumulated activity against established physical-activity recommendations (American College of Sports Medicine). Thirteen children met the device-implemented recommendation and were classified as active; the remaining 12 were classified as inactive. All participants completed an individualized two-week inpatient programme of robot-assisted gait training at the Rehabilitation Center for Children, Wiktor Dega Orthopaedic and Rehabilitation Clinical Hospital, Poznań. Data collection was conducted between November 2020 and June 2023. Data were not recorded during structured therapy sessions in order to preserve patient comfort during physiotherapy and minimise the risk of device damage. 

Assessments and data collection

Walking endurance was assessed using the 6MWT, performed on the first and final day of the rehabilitation stay in accordance with the standardised protocol described by Agarwala and Salzman [9]; the test demonstrates good test-retest reliability (r = 0.87) and a minimal clinically important difference (MCID) of 4-28 m for children with CP at GMFCS levels I-II [10-12]. Results of the 6MWT were reported in metres, rounded down to the nearest metre. The primary derived parameters were: wear-time-normalised step rate (steps per hour of wear time), caloric expenditure per hour of wear time (kcal/h), percentage of wear time spent in physical activity, and percentage of wear time spent sedentary.

Spatiotemporal gait parameters were obtained from instrumented gait analysis performed at the Gait and Motion Analysis Laboratory at Poznań University of Medical Sciences. Assessments were conducted in close temporal proximity to the rehabilitation admission and discharge dates, either immediately before or after the inpatient stay, but not within the scheduled rehabilitation period itself. Motion capture was performed using an optoelectronic system (Vicon; Oxford Metrics PLC, Yarnton, Oxfordshire, United Kingdom) comprising six Bonita 3 cameras and two Vero 2.2 cameras, operating at a sampling rate of 120 Hz and controlled via Vicon Nexus 2.11 software. Marker-based motion capture was performed using the lower body Plug-in Gait model [13]. Participants were instructed to walk at their own comfortable pace. Bilateral spatiotemporal parameters were extracted and averaged across left and right sides for each participant.

Statistical analyses

Statistical analyses were performed using Statistica version 14 (TIBCO Software Inc., Palo Alto, California, United States). Normal distribution of variables was verified with the Shapiro-Wilk test (all groups: W > 0.93, p > 0.05). Changes in 6MWT distance between the first (T1) and second (T2) assessments were analysed using a paired-sample t-test. Between-group differences in wear-time-normalised physical activity metrics (active vs inactive) were assessed using the Mann-Whitney U test. Correlations between normalised accelerometer parameters and 6MWT change were assessed using Pearson's correlation coefficient; the Spearman rank correlation coefficient was used to assess the relationship between baseline (T1) 6MWT distance and rehabilitation-induced change within subgroups. Positive r values indicate a positive correlation (higher PA associated with greater improvement); negative r values indicate a negative (inverse) correlation. Correlation magnitude was interpreted as: |r| < 0.20 negligible, 0.20-0.39 weak, 0.40-0.59 moderate, 0.60-0.79 strong, ≥0.80 very strong [14]. Effect size for paired comparisons was calculated as Cohen's d_z_ [15]. The statistical significance threshold was set at α = 0.05.

No formal a priori sample size calculation was performed, as the study was designed as an exploratory observational study. To control for multiple comparisons across exploratory tests, a family-wise error rate (FWER) correction was applied using the Holm sequential method. Correction was applied within families of related tests (between-group physical-activity differences; physical-activity-Δ6MWT correlations). The spatiotemporal gait comparisons were treated as a separate exploratory set and are reported without correction for multiple comparisons.

## Results

Descriptive characteristics and physical activity data for the 25 participants included in the analysis are presented in Table 1.

**Table 1:** 6MWT and physical activity assessment results Between-group comparisons (Active vs Inactive) were performed using the Mann-Whitney U test. Dash (-) indicates that a between-group statistical comparison was not applicable for this parameter. Wear time was not significantly different between groups (U = 67, p = 0.568); values are derived from total accelerometer wear time (range 23–54 h for the entire group). Δ6MWT distance (%) was calculated as $\left( \frac{T_2 - T_1}{T_1} \right) \times 100$, where T1 = baseline (first day) and T2 = post-rehabilitation (final day). Cohen's dz reflects the effect size for the within-group pre–post comparison and was calculated as the mean of individual paired differences (T2 − T1) divided by the standard deviation of those differences. Activity time (% of wear time) is the proportion of total valid accelerometer wear time during which the participant was classified as physically active by the device algorithm; Sedentary time (% of wear time) is the complementary proportion classified as sedentary (Activity time + Sedentary time = 100%). 6MWT: six-minute walk test * p < 0.05.

Parameter	Entire group (N=25), mean ± SD	Active (n=13), mean ± SD	Inactive (n=12), mean ± SD	Test (U)	p (Active vs Inactive)
Wear time (h)	33.95 ± 7.56	32.76 ± 5.88	35.24 ± 9.13	U=67	0.568
Average activity duration (min)	505 ± 174	588 ± 149	416 ± 157	U=122	0.018*
Average number of steps	13251 ± 6526	17997 ± 3805	8109 ± 4656	U=150	0.0001*
Calories burned during activity (kcal)	1102 ± 398	1283 ± 393	906 ± 309	U=122	0.018*
6MWT T1 distance (m)	397 ± 108	345 ± 103	453 ± 87	U=32	0.013*
6MWT T2 distance (m)	416 ± 105	370 ± 113	466 ± 69	U=34	0.017*
Δ6MWT distance (m)	19 ± 42	24 ± 34	14 ± 49	U=92	0.463
Δ6MWT distance (%)	6 ± 11	7 ± 9	4 ± 12	U=95	0.370
Cohen’s dz (Δ6MWT) [95% CI]	0.46 [0.05, 0.87]	0.70 [0.09, 1.31]	0.27 [−0.31, 0.85]	–	–
Activity time (% of wear time)	25.0 ± 8.2	30.2 ± 7.4	19.4 ± 4.5	U=143	0.0005*
Caloric expenditure (kcal/h)	33.3 ± 11.8	39.7 ± 11.6	26.3 ± 7.3	U=123	0.016*
Steps (steps/h)	403 ± 205	559 ± 125	234 ± 121	U=154	<0.0001*
Sedentary time (% of wear time)	75.0 ± 8.2	69.8 ± 7.4	80.6 ± 4.5	U=13	0.0005*

Between-group comparisons revealed statistically significant differences in all normalised physical activity metrics (Mann-Whitney U test): caloric expenditure (U = 123; p = 0.016), step rate (U = 154; p < 0.0001), and sedentary time (U = 13; p = 0.0005). The active subgroup demonstrated higher caloric expenditure and step rate and lower sedentary time.

**Figure 1:**
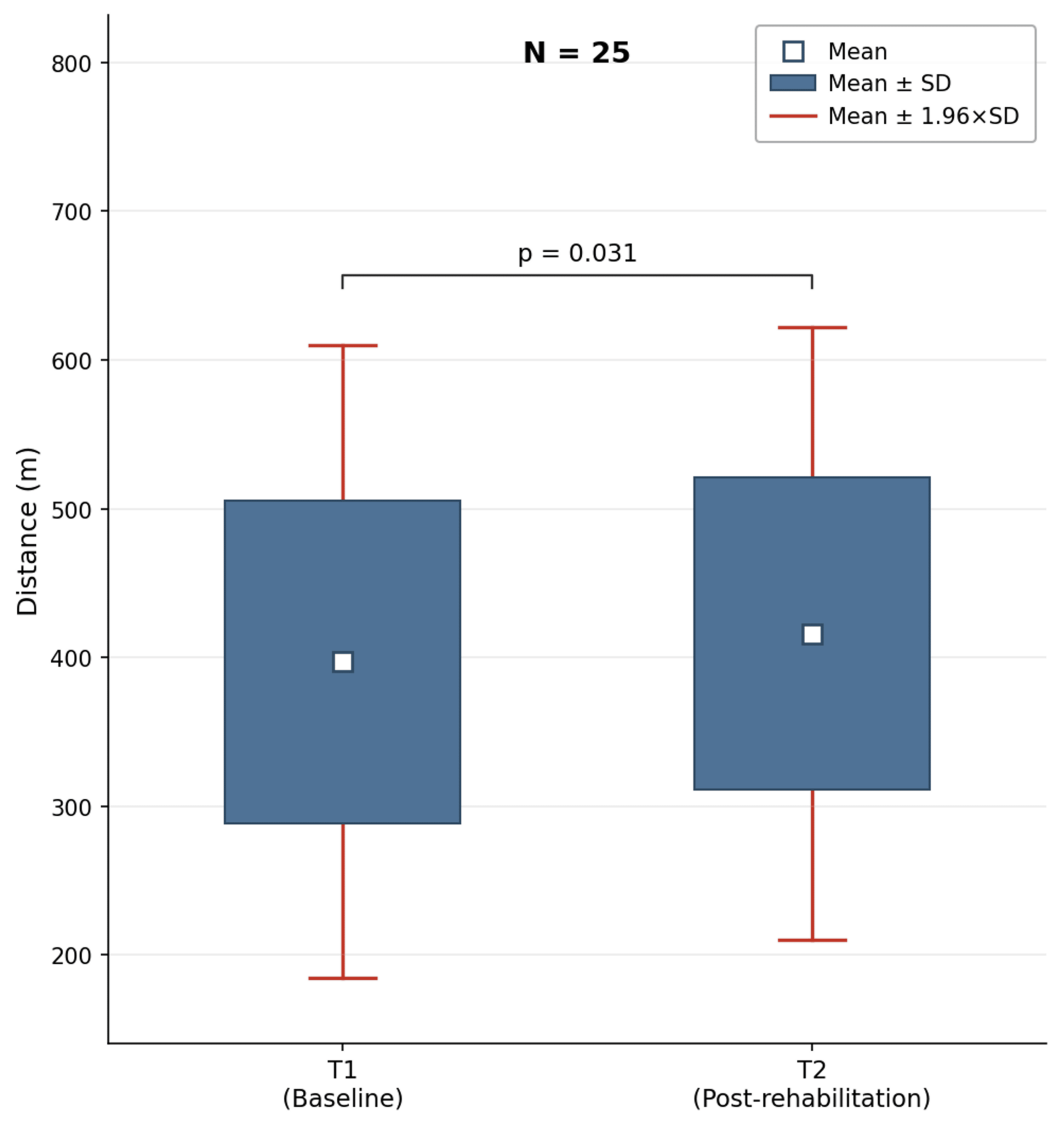
Six-minute walk test (6MWT) distances at baseline and post-rehabilitation in the entire study group (N = 25) T1 = first day of the two-week inpatient rehabilitation programme (baseline); T2 = final day of the two-week inpatient rehabilitation programme (post-rehabilitation). Boxes represent mean ± SD; whiskers represent mean ± 1.96×SD; the central square marker denotes the mean. A statistically significant improvement in 6MWT distance was observed (paired t-test: t(24) = 2.29, p = 0.031).

An exploratory analysis of the ceiling effect within the inactive subgroup revealed a strong negative Spearman’s correlation between baseline (T1) 6MWT distance and subsequent rehabilitation-induced change (T2) (ρ = −0.80; p = 0.002), indicating that children with lower baseline (T1) walking endurance benefited more from the rehabilitation programme. A statistically significant improvement in 6MWT distance was observed in the entire group (baseline (T1): 397 m; post-rehabilitation (T2): 416 m; t (24) = 2.29; p = 0.031; Figure 1).

**Figure 2:**
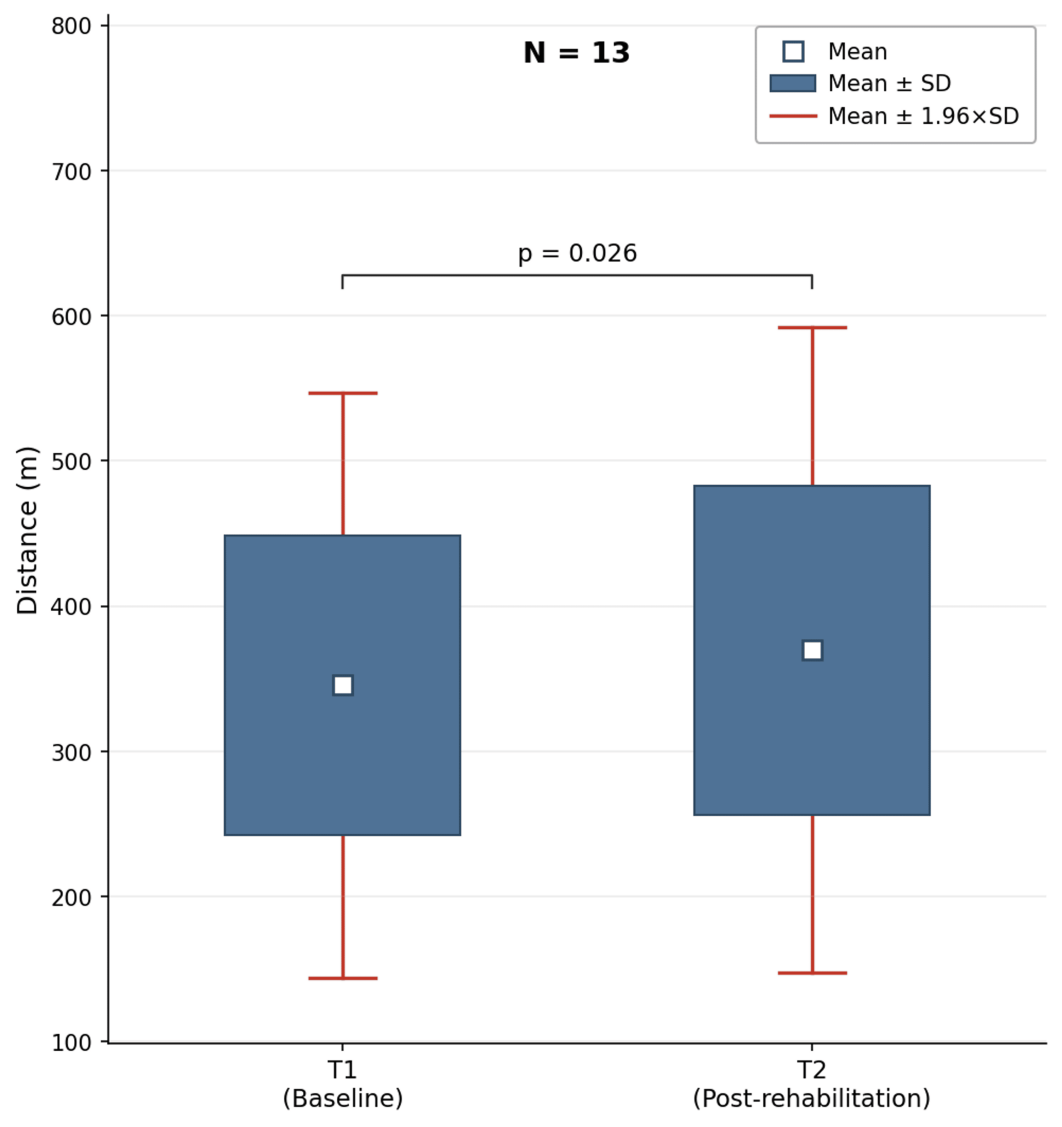
Six-minute walk test (6MWT) distances at baseline and post-rehabilitation in the physically active subgroup (N = 13) T1 = first day of the two-week inpatient rehabilitation programme (baseline); T2 = final day of the two-week inpatient rehabilitation programme (post-rehabilitation). Boxes represent mean ± SD; whiskers represent mean ± 1.96×SD; the central square marker denotes the mean. A statistically significant improvement in 6MWT distance was observed (paired t-test: t(12) = 2.54, p = 0.026). No statistically significant improvement was observed in the inactive subgroup (t(11) = 0.95, p = 0.363).

In the active subgroup (T1: 345 m; T2: 370 m; t (12) = 2.54; p = 0.026; Figure 2). No significant change was found in the inactive subgroup (t (11) = 0.95; p = 0.363). In the inactive subgroup, the 95% CI for the effect size included zero (Cohen’s d_z_ = 0.27, 95% CI −0.31 to 0.85), indicating that the non-significant change reflects imprecision under a small sample rather than a confirmed absence of effect.

After Holm correction, no statistically significant correlations were observed between any of the wear-time-normalised physical activity parameters and 6MWT change in any of the analysed groups (Table 2).

**Table 2:** Pearson’s correlation coefficients (r) and statistical significance (p) between wear-time-normalised physical activity parameters and Δ6MWT distance Normalised metrics derived from total accelerometer wear time (range 23–54 hours); All confidence intervals include zero. 6MWT: six-minute walk test

Correlation	Entire group (N=25), r; p [95%CI]	Active (n=13), r; p [95%CI]	Inactive (n=12), r; p [95%CI]
Steps/h × Δ6MWT (m)	r=0.066; p=0.755; [−0.34, 0.45]	r=−0.104; p=0.734; [−0.62, 0.47]	r=−0.042; p=0.896; [−0.60, 0.54]
Steps/h × Δ6MWT (%)	r=0.080; p=0.705; [−0.33, 0.46]	r=−0.108; p=0.727; [−0.62, 0.47]	r=+0.024; p=0.941; [−0.56, 0.59]
kcal/h × Δ6MWT (m)	r=−0.011; p=0.958; [−0.40, 0.39]	r=−0.040; p=0.897; [−0.58, 0.52]	r=−0.211; p=0.510; [−0.70, 0.41]
kcal/h × Δ6MWT (%)	r=−0.031; p=0.884; [−0.42, 0.37]	r=−0.081; p=0.792; [−0.61, 0.49]	r=−0.211; p=0.510; [−0.70, 0.41]
Activity % × Δ6MWT (m)	r=0.018; p=0.934; [−0.38, 0.41]	r=−0.127; p=0.680; [−0.63, 0.46]	r=0.108; p=0.738; [−0.50, 0.64]
Activity % × Δ6MWT (%)	r=0.021; p=0.920; [−0.38, 0.41]	r=−0.118; p=0.702; [−0.63, 0.46]	r=0.119; p=0.713; [−0.49, 0.65]

To investigate whether spatiotemporal gait characteristics underlie the divergence in baseline (T1) walking endurance between subgroups, gait parameters were compared between the active and inactive groups. Results are presented in Table 3. The active subgroup demonstrated significantly lower walking speed (0.738 ± 0.230 vs. 0.934 ± 0.139 m/s; p = 0.022), shorter step length (0.430 ± 0.126 vs. 0.516 ± 0.045 m; p = 0.041), and shorter stride length (0.855 ± 0.255 vs. 1.030 ± 0.098 m; p = 0.036) compared with the inactive subgroup. Foot off percentage was higher in the active group than in the inactive group (66.8 ± 3.8 vs. 63.1 ± 3.1%; p = 0.034), indicating a prolonged stance phase. Foot-off denotes the point in the gait cycle at which the foot leaves the ground (the end of stance); a higher foot-off percentage therefore reflects a stance phase occupying a greater proportion of the gait cycle, with a correspondingly shorter swing phase [16]. Cadence did not differ significantly between groups.

**Table 3:** Spatiotemporal gait parameters (mean ± SD) by group Between-group comparisons (Active vs Inactive) were performed using the independent-samples t-test (df = 23). Values derived from instrumental gait analysis performed within 30 days of the PA monitoring period. Between-group gait comparisons were not corrected for multiple comparisons and are exploratory. * p < 0.05

Parameter	Entire group (N = 25), mean ± SD	Active (n = 13), mean ± SD	Inactive (n = 12), mean ± SD	t (df = 23)	p
Walking Speed (m/s)	0.832 ± 0.213	0.738 ± 0.230	0.934 ± 0.139	−2.457	0.022*
Cadence (steps/min)	106.5 ± 13.6	104.8 ± 16.5	108.5 ± 10.0	−0.887	0.384
Step Length (m)	0.471 ± 0.104	0.430 ± 0.126	0.516 ± 0.045	−2.165	0.041*
Stride Length (m)	0.939 ± 0.212	0.855 ± 0.255	1.030 ± 0.098	−2.227	0.036*
Foot Off (%)	65.0 ± 3.9	66.8 ± 3.8	63.1 ± 3.1	+2.254	0.034*

## Discussion

The present study examined the relationship between habitual physical activity, quantified by accelerometer over a 72-hour free-living period, and change in walking endurance following a two-week inpatient rehabilitation programme in children with CP at GMFCS II. The primary finding was a statistically significant and clinically meaningful improvement in 6MWT distance in the entire group and the active subgroup, consistent with the growing body of evidence supporting structured inpatient rehabilitation as an effective intervention for improving functional mobility in this population [11,12].

The magnitude of improvement in both groups fell within or approached the reported MCID for the 6MWT in ambulatory children with CP [17], with the active subgroup achieving a medium-to-large effect size. Despite these gains, both groups remained substantially below published normative 6MWT distances for typically developing peers of the same age [18], underscoring the persistent functional limitation in this population. The inactive subgroup did not demonstrate a significant improvement, which may reflect either true non-responsiveness to the rehabilitation programme or insufficient statistical power given the small subgroup size. These findings are consistent with a previous study [7], which reported a significant improvement in the active group (p = 0.04) but not in the inactive group (p = 0.25) using the same accelerometer protocol in a smaller post-exclusion sample (n = 14) collected during the COVID-19 pandemic. Beyond enrolling a larger sample, the principal advance of the present study is the addition of instrumented three-dimensional gait analysis, absent from the earlier report, which provides a candidate biomechanical mechanism for the previously unexplained paradox: the MET-classified active subgroup showed shorter step and stride length, lower walking speed, and a proportionally longer stance phase, a pattern that may constrain timed-distance performance despite higher daily step accumulation. The larger sample additionally improves the robustness of the subgroup comparisons.

A notable and seemingly paradoxical finding was the substantially lower baseline walking endurance in the active subgroup compared with the inactive subgroup, despite higher habitual step accumulation. Although cadence did not differ significantly between subgroups (p = 0.384), walking speed, step and stride lengths, and gait phasing (a proportionally longer stance phase) differed in this exploratory analysis. The paradoxically lower timed-distance performance of the MET-classified active subgroup may therefore be better explained by these spatial and phasic characteristics (shorter, less efficient steps and a relatively longer stance) than by a cadence-based compensation strategy: comparable step frequency combined with a shorter step constrains absolute distance covered in a timed test despite higher daily step counts [19,20]. Children with mechanically efficient, longer-stride gait may achieve superior 6MWT performance while remaining below the daily MET threshold. Differences in gait characteristics between clinical and free-living conditions in this population have been reported [21]. The present spatiotemporal findings are consistent with this mechanism: spatiotemporal gait analysis revealed that the active subgroup had significantly lower walking speed, shorter step length, and shorter stride length than the inactive subgroup, suggesting that MET-classified active children exhibit less efficient gait biomechanics despite higher daily step accumulation. The absence of significant correlations between wear-time-normalised PA metrics and 6MWT change across all groups further indicates no statistically detectable association between habitual activity intensity and rehabilitation-induced walking gains, consistent with evidence from the broader CP literature [3,6].

An exploratory analysis of the inactive subgroup revealed a strong negative association between baseline walking endurance and rehabilitation-induced change, suggesting that children with greater baseline functional limitation derived greater benefit from the standardised programme. Regression to the mean cannot be excluded as a contributing factor, and this finding should be regarded as hypothesis-generating given the small subgroup size and absence of a control group.

Limitations and future recommendations

Several limitations should be acknowledged. The sample was small, and no a priori sample-size calculation was performed; the study was particularly underpowered for the subgroup, correlation, and gait-parameter analyses, in which small samples increase the risk of type II (false-negative) findings and yield imprecise effect estimates. These analyses should be regarded as exploratory. The absence of a control group precludes conclusions about whether improvements reflect the specific effect of rehabilitation or natural variation over time. Accelerometer monitoring was limited to 72 hours with mandatory device removal during sleep and bathing, and data were not recorded during therapy sessions, resulting in variable effective wear times that may not adequately capture habitual activity patterns. The study was conducted at a single centre, and the cross-sectional design of the spatiotemporal comparison precludes causal inference. The active/inactive classification relied on a proprietary, manufacturer-defined algorithm that has not been validated specifically in children with cerebral palsy and should therefore be interpreted as a pragmatic, device-derived categorisation.

Future research should address these limitations through larger multi-centre designs, extended accelerometer monitoring, inclusion of control groups, multi-variable modelling incorporating spatiotemporal and energy-cost parameters, and longitudinal follow-up to determine whether gait biomechanics mediate the relationship between habitual physical activity and rehabilitation outcomes.

## Conclusions

Two weeks of inpatient rehabilitation resulted in significant and clinically meaningful improvements in walking endurance in children with CP at GMFCS II. The effect was greatest in the active subgroup, reaching medium-to-large magnitude (Cohen's d_z_ = 0.70) and exceeding the MCID for this population, supporting the continued provision of structured inpatient rehabilitation for this functionally heterogeneous group. Spatiotemporal gait analysis revealed inefficient gait biomechanics in the active subgroup compared with the inactive subgroup, with significantly lower walking speed, shorter step and stride length, and prolonged stance phase. These differences may help explain the paradoxical inverse relationship between MET-based activity classification and baseline walking endurance observed in this population.

The absence of significant correlations between wear-time-normalised accelerometer parameters and 6MWT improvement in any group indicates that habitual PA intensity does not determine the magnitude of rehabilitation benefit, and that accelerometer-based monitoring and clinical walking tests reflect independent dimensions of functional motor ability in this population. Subgroup comparisons and correlation analyses were exploratory; the pre-specified primary endpoint, improvement in 6MWT distance, remains the primary basis for inference.
